# Cross-Polarized Surface-Enhanced Infrared Spectroscopy by Fano-Resonant Asymmetric Metamaterials

**DOI:** 10.1038/s41598-017-03545-8

**Published:** 2017-06-09

**Authors:** Atsushi Ishikawa, Shuhei Hara, Takuo Tanaka, Yasuhiko Hayashi, Kenji Tsuruta

**Affiliations:** 1 Department of Electrical and Electronic Engineering, Okayama University, Okayama, Okayama, 700-8530 Japan; 2 Metamaterials Laboratory, RIKEN, Wako, Saitama, 351-0198 Japan; 3 Innovative Photon Manipulation Research Team, RIKEN Center for Advanced Photonics, Wako, Saitama, 351-0198 Japan; 4 Department of Chemical Science and Engineering, Tokyo Institute of Technology, Yokohama, Kanagawa 226-8503 Japan

## Abstract

Plasmonic metamaterials have overcome fundamental limitations in conventional optics by their capability to engineer material resonances and dispersions at will, holding great promise for sensing applications. Recent demonstrations of metamaterial sensors, however, have mainly relied on their resonant nature for strong optical interactions with molecules, but few examples fully exploit their functionality to manipulate the polarization of light. Here, we present cross-polarized surface-enhanced infrared absorption (SEIRA) by the Fano-resonant asymmetric metamaterial allowing for strong background suppression as well as significant field enhancement. The metamaterial is designed to exhibit the controlled Fano resonance with the cross-polarization conversion property at 1730 cm^−1^, which spectrally overlaps with the C=O vibrational mode. In the cross-polarized SEIRA measurement, the C=O mode of poly(methyl methacrylate) molecules is clearly observed as a distinct dip within a Fano-resonant transmission peak of the metamaterial. The vibrational signal contrast is then improved based on the cross-polarized detection scheme where only the light interacting with the metamaterial-molecular coupled system is detected by totally eliminating the unwanted background light. Our metamaterial approach achieves the zeptomole sensitivity with a large signal-to-noise ratio in the far-field measurement, paving the way toward the realization of ultrasensitive IR inspection technologies.

## Introduction

Controlling the polarization state of light is of great importance in advanced optics for fully utilizing valuable information of electromagnetic waves^1^. Polarized illumination and measurement are simple yet powerful techniques to maximize signal contrast against the background, hence widely used in diverse fields including optical metrology, communication, and display technology^2^. In such optical systems, the polarization conversion or rotation has been achieved by birefringent or gyrotropic materials, although they are naturally suffering from several drawbacks of extended sizes and limited bandwidth^3^. Plasmonic metamaterials, on the other hand, have overcome fundamental limitations in conventional optics by engineering the resonant and dispersion properties of metallodielectric nanostructures^4–6^, bringing unusual optical phenomena and functionalities into reality^7–15^. An example of their potential applications includes a metamaterial-based polarimetric device to control the polarization in a desired manner^16–18^, and such a new capability may offer a powerful platform for novel sensing applications^19, 20^.

Surface-enhanced infrared absorption (SEIRA) by plasmonic nanostructures has played a central role in material and life science for direct analysis of molecular functional groups^21^. Despite of the long history of SEIRA research, there is still high demand for ultrasensitive detection in sophisticated applications, such as environmental monitoring and breath diagnosis. Different from conventional metal island films, tailored plasmonic nanoantennas were proposed to improve the spatial and spectral mode overlapping between the plasmons and molecular vibrations^22–28^. Fine spectral tuning was also demonstrated by using engineered IR metamaterials, where the steep dispersion arising from the Fano resonance led to a large spectral selectivity^29–32^. Although atto/zeptomole sensitivity of SEIRA has been achieved so far, these approaches have mainly relied on their resonant nature for strong optical interactions with molecules, but few examples fully exploit their novel optical functionalities. On the other hand, our recent demonstration of the metamaterial IR absorber proved that background suppression in SEIRA could be an alternative approach to gain a large signal contrast for better sensitivity^33^.

Here, we propose cross-polarized SEIRA by the Fano-resonant asymmetric metamaterial (FRAM) allowing for strong background suppression as well as significant field enhancement. Specifically, we experimentally demonstrate the resonant coupling of the Fano mode of the metamaterial and IR vibrational mode of polymer molecules. By fully exploiting the polarization conversion property of the metamaterial, the cross-polarized detection scheme is demonstrated where only the light interacting with the metamaterial-molecular coupled system is detected by totally eliminating the unwanted background light. Different from conventional hot-spot engineering to improve the near-field enhancement, the vibrational signal contrast against the background is improved for better sensitivity of SEIRA. Our metamaterial approach achieves the zeptomole sensitivity with a large signal-to-noise (S/N) ratio in the far-field measurement, thus further lowering the detection limit of direct IR absorption spectroscopy.

**Figure 1:**
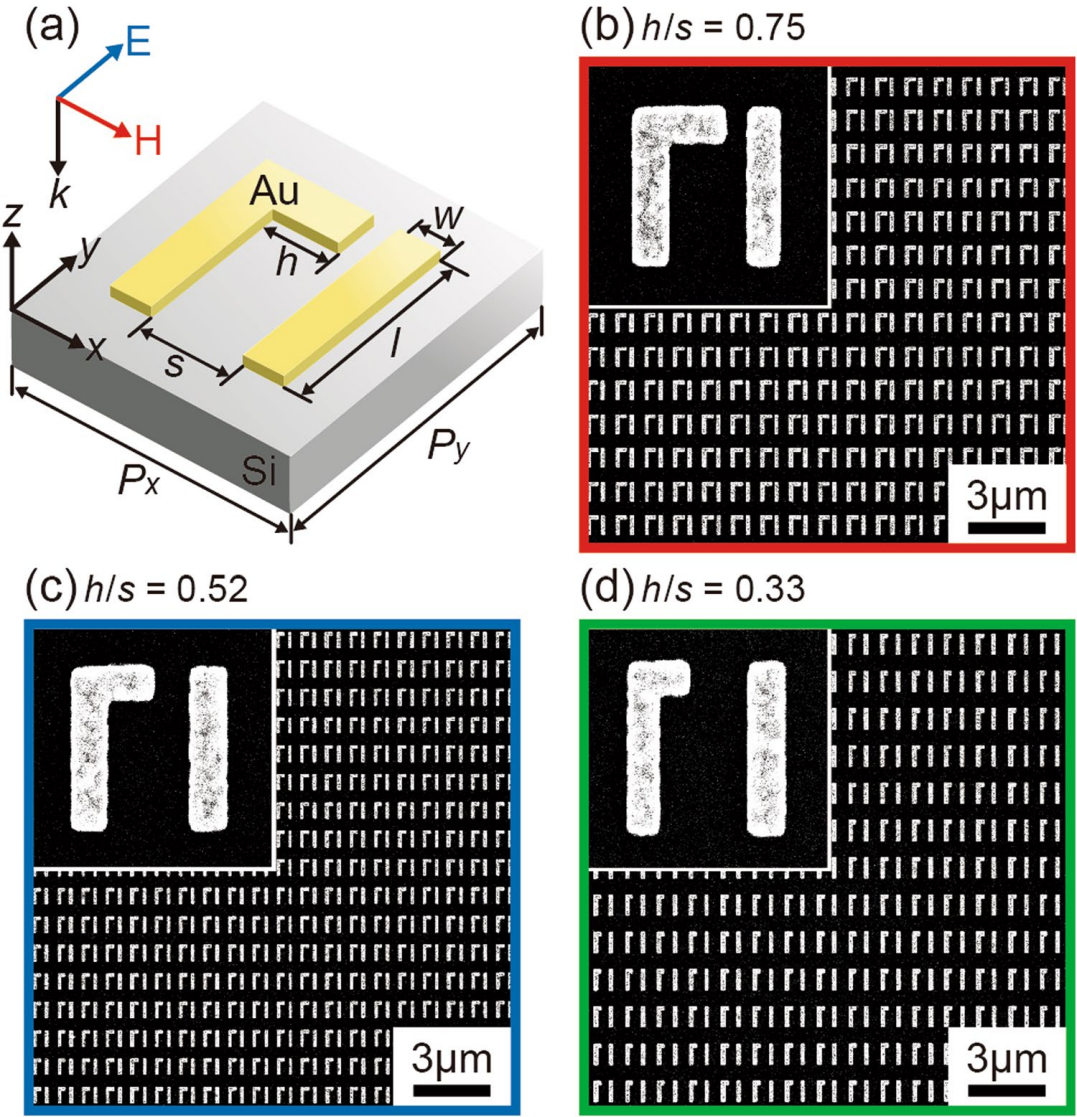
Design and fabrication of metamaterials. (**a**) Schematic unit cell of a metamaterial on a Si substrate consisting of an Au nano-rod pair with a horizontal coupling antenna to break the structural symmetry. The surface structure was designed to exhibit the pronounced Fano resonance at 1730 cm^−1^, which spectrally overlapped with the C=O vibrational mode. (**b**–**d**) SEM images of the fabricated metamaterials with different antenna lengths of *h*/*s* = 0.75 (red), 0.52 (blue), and 0.33 (green) to tune the excitation of the Fano resonance. To match the Fano-resonant frequencies of all the metamaterials even with different antenna lengths, other geometry parameters were carefully designed (see the Methods section).

Figure 1(a) illustrates the schematic of the fabricated FRAM consisting of an Au nano-rod pair with a horizontal coupling antenna to break the structural symmetry^30^. As shown in the scanning electron microscopy (SEM) images of Fig. 1(b)–(d), three types of the FRAMs with different antenna lengths were fabricated with the ratio of *h*/*s* = 0.75 (red), 0.52 (blue), and 0.33 (green) to tune the excitation of the Fano resonance (see the Methods section). Even with different antenna lengths, each FRAM was carefully designed to exhibit the same Fano resonant frequency of 1730 cm^−1^, which spectrally overlapped with the C=O vibrational mode (*ω*_C=O_).

**Figure 2:**
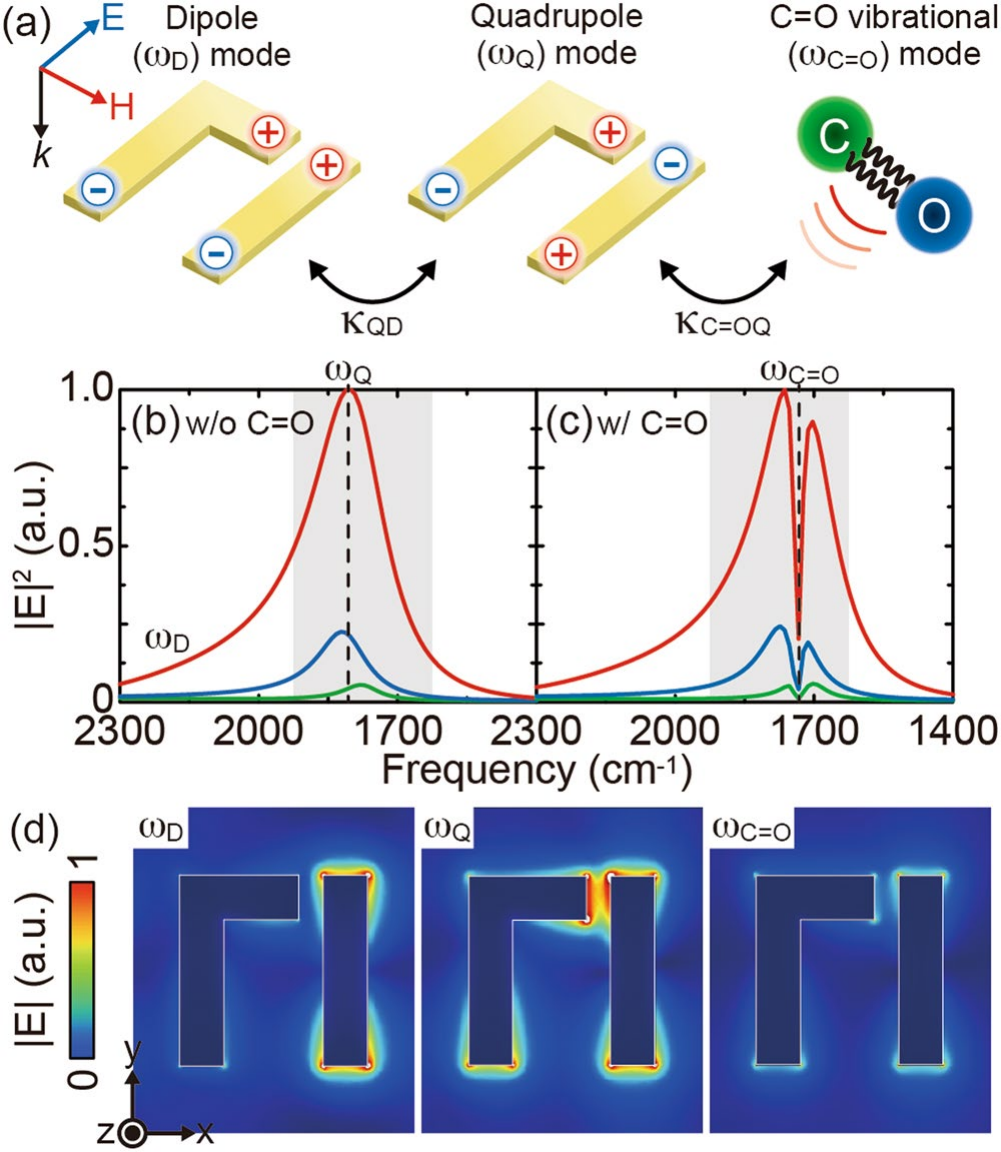
Near-field responses of a metamaterial-molecular coupled system. (**a**) Schematic of the mode interaction. The interference between the dipole (*ω*_D_) and quadrupole (*ω*_Q_) plasmon modes produces the Fano resonance, which resonantly coupled with the C=O vibrational mode (*ω*_C=O_). Numerically simulated |E|^2^ spectral responses at the gap center of the metamaterials (**b**) without and (**c**) with the C=O mode. (**d**) Corresponding |E| distributions at *ω*_D_ and *ω*_Q_ in (**b**) and at *ω*_C=O_ in (**c**) for *h*/*s* = 0.75 (red). At the Fano resonance (*ω*_Q_), the y-polarized incident electric field is highly localized within the gap, inducing strong gap plasmon to re-radiate the x-polarized light. The C=O mode strongly quenches the Fano resonance by disturbing the sensitive mode interference, translating into distinct far-field spectral response.

Figure 2(a) is the schematic of the underlying mode interaction in a metamaterial-molecular coupled system. For the y-polarized incident wave, a dipole plasmon mode (*ω*_D_) is excited in the FRAM associated with in-phase charge oscillation (left). At the same time, a quadrupole mode (*ω*_Q_) with out-of-phase charge oscillation (center) is also excited, due to the symmetry breaking induced by the coupling antenna. Once the low-Q dipole and high-Q quadrupole modes are spectrally overlapped and interfered, the dispersive Fano resonance is formed with significant field enhancement within the gap^34^. Additional coupling of the C=O vibrational mode with the FRAM disturbs the sensitive mode interference, thereby strongly modifying both the near- and far-field spectral responses. Note that such a detection scheme is not limited to the C=O mode, but can be applied for other kinds of molecular vibrations, since it is based on a generalized model of linearly-coupled harmonic oscillators^33^.

To demonstrate the aforementioned mode interaction and the resultant optical responses of the system, a set of numerical simulations based on finite element method (FEM) was carried out (see the Methods section). Fig. 2(b) shows the numerically simulated |E|^2^ spectral responses at the gap center of the bare FRAMs with *h*/*s* = 0.75 (red), 0.52 (blue), and 0.33 (green), while Fig. 2(c) is the same ones, but for the FRAMs covered with a 50-nm thick poly(methyl methacrylate) (PMMA) layer containing the C=O bond in the monomer unit. The corresponding |E| distributions at *ω*_D_, *ω*_Q_, and *ω*_C=O_ for *h*/*s* = 0.75 (red) are also shown in Fig. 2(d). At the Fano resonance, the y-polarized incident electric field is highly localized and strongly enhanced within the gap, arising from the quadrupole nature of the Fano resonance *ω*_Q_ in Fig. 2(b) and (d). The field enhancement becomes even stronger by increasing the antenna length, thereby increasing the coupling strength between the dipole and quadrupole modes (к_QD_). Since the excited gap plasmon oscillates along the x-axis, the polarization of the y-polarized incident light is partially rotated by 90°, thus the FRAM exhibits the cross-polarization conversion property. Such a polarization property as well as the significant field enhancement can be obtained only at the Fano resonance, but not at the dipole resonance *ω*_D_ in Fig. 2(b) and (d). Once the PMMA molecules are attached onto the FRAM, on the other hand, the C=O mode strongly quenches the Fano resonance by disturbing the sensitive mode interference *ω*_C=O_ in Fig. 2(c) and (d). These polarized local responses are then translated into distinct far-field spectral responses for the high-sensitive cross-polarized SEIRA measurement.

**Figure 3:**
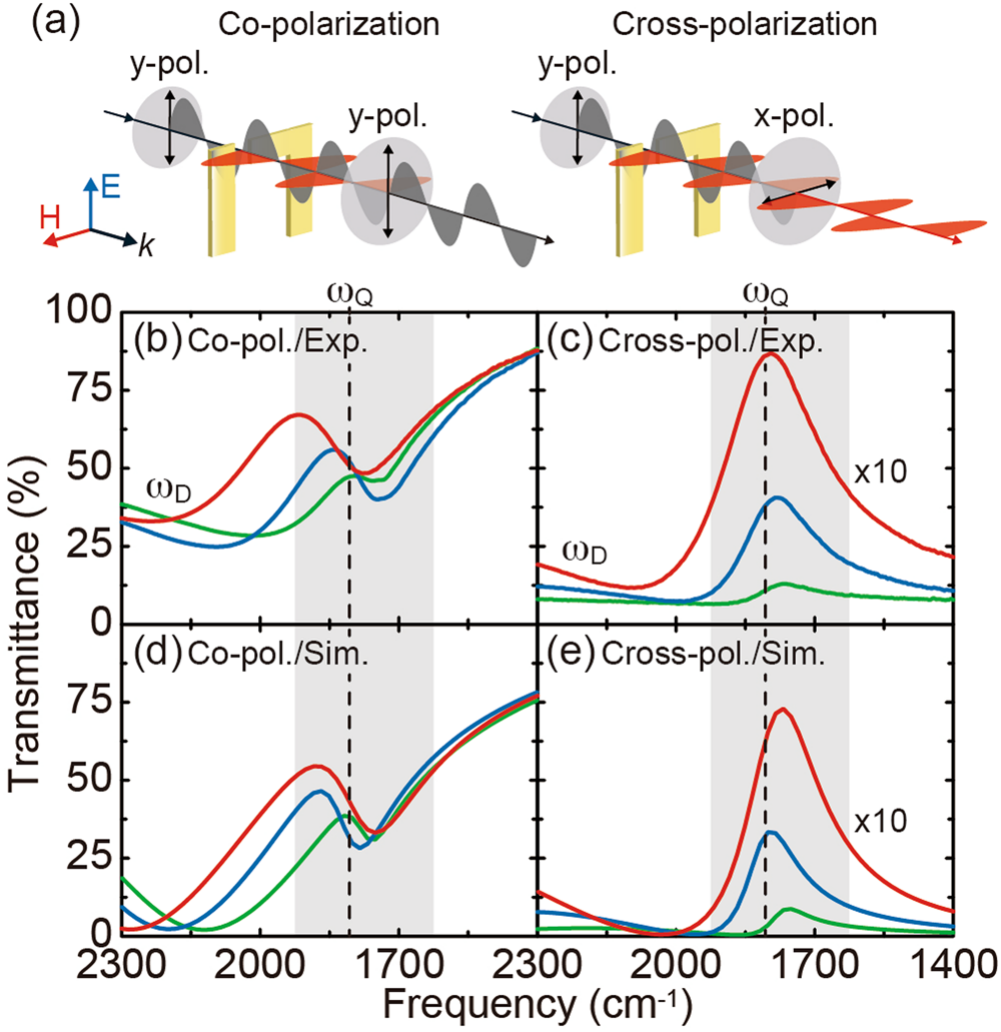
IR characterization of metamaterials. (**a**) Experimental setups of FT-IR transmission measurement for the co- and cross-polarizations. Experimentally measured transmission spectra of the metamaterials for the (**b**) co- and (**c**) cross-polarizations. (**d**, **e**) Corresponding numerical simulations, which well reproduced the experimental results qualitatively and quantitatively. The excitation of the Fano resonance in the shaded region is manifested as a transmission dip for the co-polarization, but as a transmission peak for the cross-polarization. The spectra in (**c**) and (**e**) have been multiplied by 10 for clarify.

The transmission properties of the FRAMs were experimentally characterized by using a Fourier-Transform Infrared Spectrometer (FT-IR) equipped with a polarized infrared microscope (see the Methods section). As shown in Fig. 3(a), two identical polarizers were aligned before and behind the sample to perform the co- and cross-polarized measurements. Figure 3(b) shows the measured co-polarized transmission spectra of the FRAMs with different antenna lengths. The asymmetric Fano line-shapes are clearly observed at 1730 cm^−1^ and become pronounced by increasing the antenna length for stronger mode coupling. Figure 3(c) shows the same ones, but for the cross-polarization where the excitation of the Fano resonance is manifested as a transmission peak at 1730 cm^−1^. At the Fano resonance *ω*_Q_ in Fig. 2(d), a strong gap plasmon with dipole orientation along the x-axis is excited to re-radiate the x-polarized light into forward and backward directions. Since the y-polarized incident light is eliminated by the polarizer behind the sample, only the x-polarized forward radiation from the gap is transmitted and selectively detected, thus giving a single peak at 1730 cm^−1^ in the cross-polarized transmission. These experimental results were well reproduced by the corresponding numerical simulations, as shown in Fig. 3(d) and (e). Note that the cross-polarized measurement selectively extracts the light interacting with the metamaterial-molecular coupled system by totally eliminating the unwanted background light; therefore, the signal-to-background (S/B) ratio is greatly enhanced to improve the sensitivity of the SEIRA measurement^33^. Although the efficiency of the cross-polarization conversion is currently up to 9% in this study, this can be improved by optimizing the metamaterial design^17, 35–38^.

**Figure 4:**
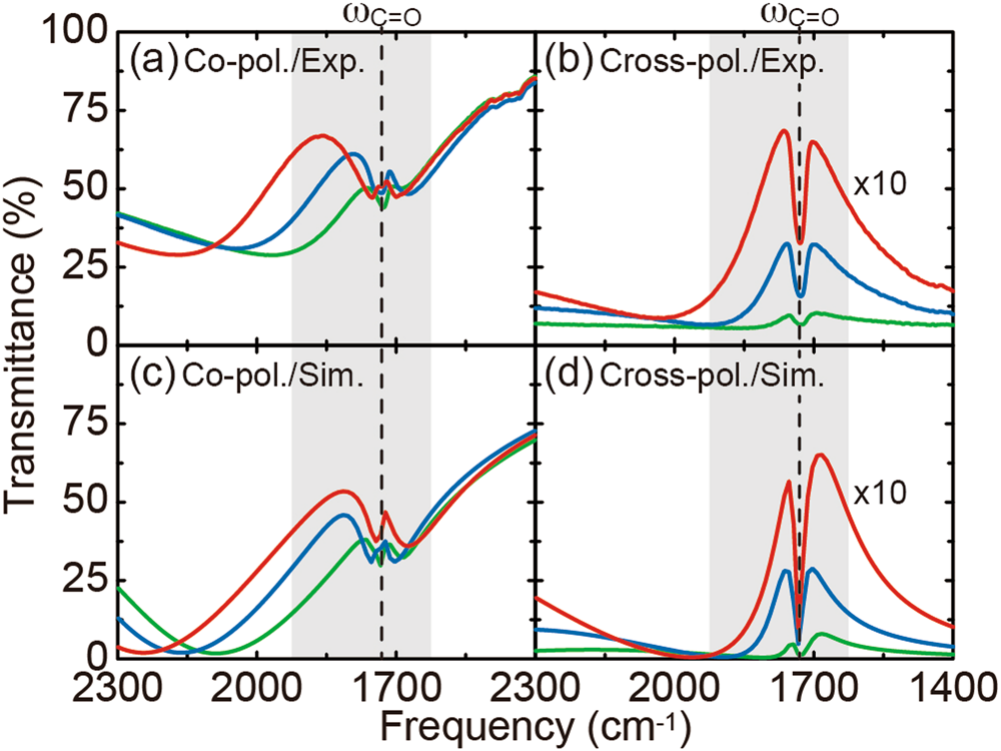
Polarized metamaterial-enhanced IR absorption. Experimentally measured transmission spectra of a 50-nm thick PMMA film on the metamaterials for the (**a**) co- and (**b**) cross-polarizations. (**c**,**d**) Corresponding numerical simulations, which well reproduced the experimental results qualitatively and quantitatively. The resonant coupling of the Fano and C=O modes at *ω*_C=O_, indicated by the dashed lines, results in an anti-resonant peak (dip) for the co- (cross-) polarization within the Fano-line shapes of the metamaterials. The cross-polarized detection scheme selectively extracts the light interacting with the metamaterial-molecular coupled system, thus dramatically improving the vibrational signal contrast compared to the conventional co-polarized case. The spectra in (**b**) and (**d**) have been multiplied by 10 for clarify.

Based on the cross-polarization conversion property achieved by the FRAM, we explored the cross-polarized SEIRA by utilizing the resonant coupling between the Fano mode of the FRAM and IR vibrational modes of molecules. In the SEIRA measurement, PMMA containing the C=O bond in the monomer unit was used as a test specimen and its C=O mode at 1730 cm^−1^ was detected. PMMA with a solid content of 2.0% in an anisole solvent was uniformly spin-coated onto the bare FRAM surface with a thickness of 50 nm (see the Methods section). Figure 4(a) shows the measured co-polarized transmission spectra of the PMMA-coated FRAMs. The resonant coupling occurs between the Fano and C=O modes, producing a clear anti-resonant peak within the Fano line-shape of the FRAMs. For the cross-polarization case in Fig. 4(b), on the other hand, a distinct anti-resonant dip appears within a transmission peak of the FRAMs. Since the detected signal in the cross-polarized SEIRA comes only from the metamaterial-molecular coupled system, the signal contrast, i.e., the ratio of vibrational signal to total one, is dramatically improved compared to the conventional co-polarized case. By increasing the antenna length, thereby increasing the excitation of the Fano resonance, the signal contrast becomes even more enhanced for better sensitivity. Fig. 4(c) and (d) show the corresponding numerical results, which well reproduce the experimental ones both qualitatively and quantitatively. In the metamaterial-molecular coupled system, the C=O mode behaves as a local oscillator with a finite absorption to disturb the Fano resonance, and this modifies the far-field spectral responses for each polarization in different ways. In the case of the co-polarization Fig. 4(c), the energy absorbed by the C=O mode couples back to the FRAM through the near-field coupling, and this in turn produced an anti-resonant peak within the Fano-line shape due to the interference between the Fano and C=O modes. For the cross-polarization case Fig. 4(d), on the other hand, the C=O mode strongly quenches the gap plasmon *ω*_C=O_ in Fig. 2(c) and (d), and this results in a distinct anti-resonant dip within a transmission peak of the FRAMs.

**Figure 5:**
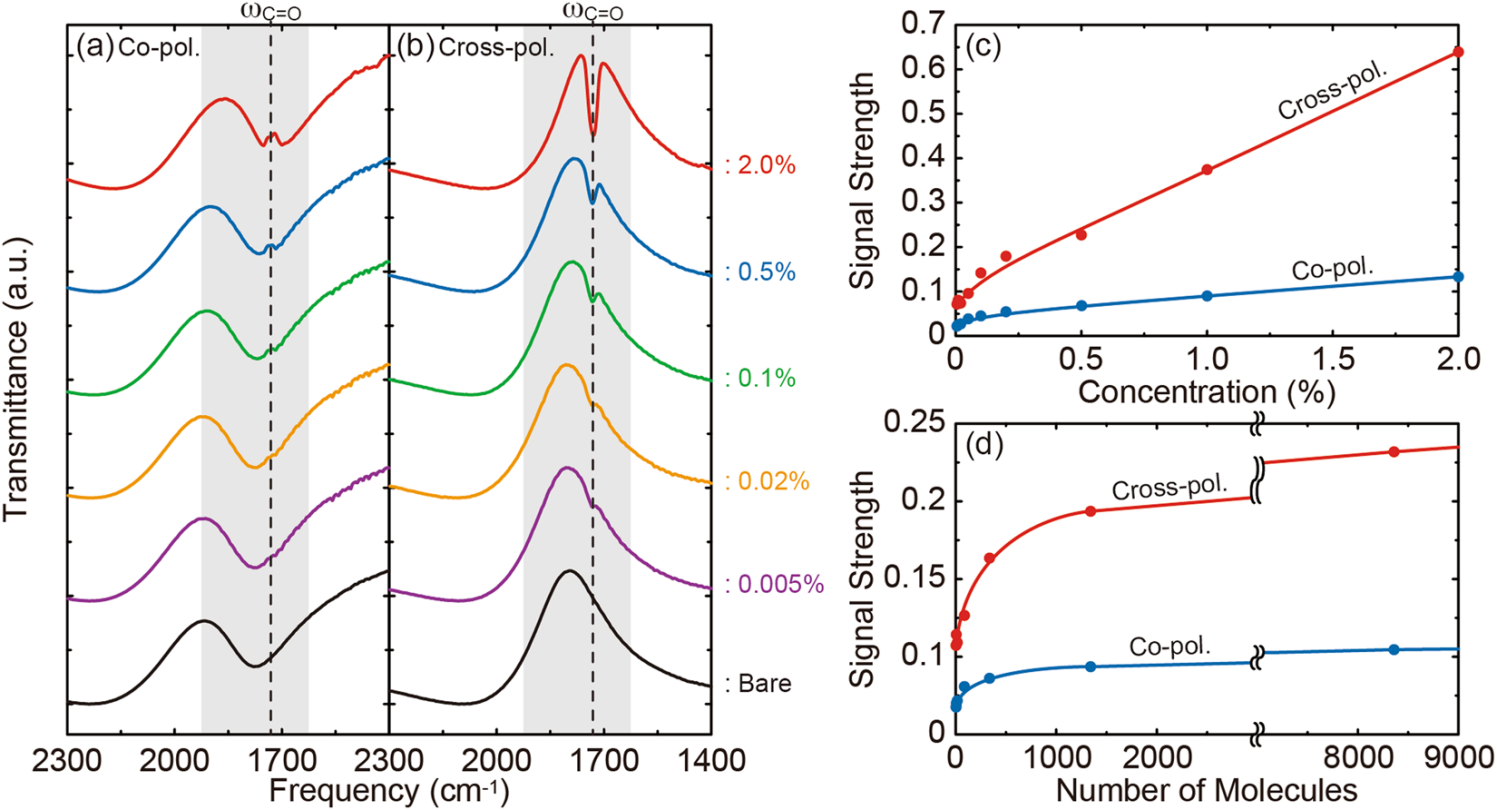
Comparison of the measured signal strengths between the co- and cross-polarizations. (**a**) Co- and (**b**) cross-polarized transmission spectra of a PMMA thin film on the metamaterials by progressively decreasing the solid content of PMMA solution. (**c**) Extracted signal strengths as the function of the PMMA concentration, and (**d**) the same ones as the function of the number of PMMA molecules within the unit cell.

To quantitatively evaluate the ultimate sensing capability of the cross-polarized SEIRA, the vibrational signal strengths for the co- and cross-polarizations were experimentally measured by progressively decreasing the solid content of PMMA solution. Figure 5(a) and (b) show the measured co- and cross-polarized transmission spectra of the PMMA-coated FRAMs *h*/*s* = 0.75 (red) for different PMMA concentrations from 2.0 to 0.005% in an anisole solvent. For better illustration to evaluate the sensitivity, the concentration dependences of the extracted signal strengths are shown in Fig. 5(c). Here, the signal strength is defined as the spectrum difference between the PMMA-coated and bare FRAMs, considering the red-shift effect due to the non-dispersive refractive index of a PMMA film^32^. By taking into account the dependence of the final film thickness on the concentration and on the spin speed^39^, Fig. 5(c) can be replotted in Fig. 5(d) as the function of the number of PMMA molecules within the unit cell. When the number of molecules decreases, the signal strengths for both polarizations become weak with similar downward tendency. Large signal reductions below ~1000 molecules are mainly due to the effect of an ultrathin film where the thickness of a PMMA film becomes much smaller than the metamaterial structures (~20 nm). The signal strength of the cross-polarized SEIRA is always more than two times larger than that of the co-polarized one, thus giving a detectable signal even for an extremely small amount of molecules. Using the PMMA molecular weight (950 × 10^3^) and solid density (1.2 g/cm^3^), the sensitivity for the 0.005% case can be estimated down to ~9.1 zeptomoles within the sample area (100 × 100 μm^2^) in the cross-polarized SEIRA measurement. These quantitative measurements clearly demonstrate the cross-polarized detection scheme with a large S/B ratio dramatically enhances the sensing capability in conventional SEIRA spectroscopy.

In conclusion, a novel spectroscopic technique based on the cross-polarized SEIRA in the Fano-resonant asymmetric metamaterials was proposed and demonstrated. The cross-polarized detection scheme by the metamaterial achieved the zeptomole sensitivity with a large signal contrast of direct IR absorption spectroscopy. The corresponding numerical simulations revealed the underlying mode interaction in the metamaterial-molecular coupled system and the cross-polarization conversion property. We showed a simple yet powerful technique to improve the performance of sensors by introducing an inexpensive cross-polarized light setup, and the same can be applied in different systems. By combining the bio-functionalized metamaterials^30^, the specificity and reliability of biosensing may be further improved, where the vibrational signal of the target biomolecule binding to the metamaterial surface is selectively detected, while the others are strongly excluded by the optical system. Hence, our metamaterial approach makes an important step of plasmonic sensing device applications for advanced IR inspection technologies.

## Methods

### Sample fabrication

A 100-nm thick electron beam (EB) resist, PMMA (MicroChem, 950-A2), was spin-coated onto a 325-µm thick double-side polished Si substrate with 2000 rpm for 55 s and baked at a 180 °C for 90 s. A two-dimensional (2D) periodic pattern of the FRAMs was exposed over an area of 100 × 100 µm^2^ by using an EB lithography system (Elionix, ELS-S50). The sample was then completed by Cr/Au (2/20 nm) deposition using a resistive heating evaporator and liftoff process with acetone. The residual EB resist was totally washed away by a UV/O_3_ ashing process, hence having no effect in the SEIRA measurement. {*P*_*x*_, *P*_*y*_, *l*, *w*, *s*, *h*} in nm were {1150, 1330, 770, 180, 400, 300} for Fig. 1(b), {1200, 1400, 800, 180, 420, 220} for Fig. 1(c), and {1240, 1450, 850, 180, 450, 150} for Fig. 1(d). The geometry parameters were carefully designed by considering the red-shift effect due to a PMMA film in the SEIRA measurement, such that the Fano and C=O modes spectrally overlapped in the PMMA-coated FRAMs.

### Numerical simulation

The electromagnetic responses of the FRAMs were calculated using the FEM software package, COMSOL Multiphysics, with *ε*_Si_ = 11.56 and the empirical value for *ε*_Au_^40^. The C=O bond in the PMMA layer was modeled as a Lorentz oscillator with the functional form: ${\varepsilon }_{PMMA}\,=\,{\varepsilon }_{b}\,+\,\frac{{f}_{m}{\omega }_{0}^{2}}{{\omega }_{0}^{2}\,-\,{\omega }^{2}\,-\,i\gamma \omega }$, where *ε*_*b*_ is the background relative permittivity, *f*_*m*_ is the reduced oscillator strength, *ω*_0_ is the C=O vibrational frequency, and *γ* is the damping constant (FWHM)^32, 41^. The parameters, *ε*_*b*_, *f*_*m*_, *ω*_0_, and *γ*, were appropriately chosen such that the oscillator model emulated the vibrational absorption of the C=O mode. Calculated transmittance was normalized by the corresponding result for the bare Si substrate to simulate the experimental condition.

### Sample characterization

A FT-IR (JASCO, FT/IR-6300FV) equipped with a polarized infrared microscope (JASCO, VIRT-3000) was used to characterize the transmittance spectra of the sample. To improve the S/N ratio of the spectra, a square aperture with an area of 100 × 100 µm^2^ was installed at the pupil plane of the microscope and a sample chamber was purged with dry nitrogen gas. Two identical polarizers (JASCO, PL-82) with a high extinction ratio of 1 × 10^−2^ over the measurement range were used for the polarized measurements. Measured spectrum was normalized by the corresponding result for the bare Si substrate to discuss only the optical responses of the FRAMs.

### PMMA layer preparation

PMMA (MicroChem, 950-A2) was uniformly spin-coated onto the bare FRAM surface with 4000 rpm for 55 s and baked at a 180 °C for 90 s. The thickness of the PMMA layer was determined by using an optical surface profiler (Zygo, NewView 7300) with a height resolution of less than 0.1 nm. 0.5% ~0.005% PMMA solutions were prepared by diluting a 2.0% PMMA solution with an anisole solvent and spin-coated in the same manner.
